# Temperature changes are signaled in cyanobacteria through the PipX interaction network

**DOI:** 10.3389/fmicb.2025.1688974

**Published:** 2025-11-26

**Authors:** Antonio Llop, Sirine Bibak, Trinidad Mata-Balaguer, Lorena Tremiño, Laura Fuertes-García, José L. Neira, Ray Dixon, Asunción Contreras

**Affiliations:** 1 Departamento de Fisiología, Genética y Microbiología, Universidad de Alicante, San Vicente del Raspeig, Spain; 2 IDIBE, Universidad Miguel Hernández, Elche, Alicante, Spain; 3 Instituto de Biocomputación y Física de Sistemas Complejos (BIFI), Universidad de Zaragoza, Zaragoza, Spain; 4 Department of Molecular Microbiology, John Innes Centre, Norwich, United Kingdom

**Keywords:** PII, NtcA, EngA, cold-shock signaling, *Synechococcus*, NanoBiT assay, NanoLuc

## Abstract

Cyanobacteria perform oxygenic photosynthesis and have evolved sophisticated mechanisms to adapt their metabolism to challenging environmental changes. Despite their ecological and biotechnological importance, many regulatory proteins are still uncharacterised, and their signalling networks are poorly studied in comparison to other bacterial phyla. Two small proteins, PipX, unique to cyanobacteria, and PII, widespread in bacteria and plants, are the hubs of a protein interaction network involved in carbon/nitrogen homeostasis, energy sensing, translational regulation and growth. Here we exploit the NanoBiT complementation system to demonstrate in real time that temperature affects PipX interactions with its best studied partners: the signal transduction protein PII, the global transcriptional regulator NtcA, and the ribosome-assembly GTPase EngA. While heat shock increased PipX-PII complex formation and impaired PipX-EngA and PipX-NtcA interactions, temperature downshift resulted in a decrease of all three complexes. However, during longer term acclimatization, each type of complex responded distinctively after either up- or downshifts in temperature and PipX-PII and PipX-NtcA interactions were influenced in opposite ways. Altogether the results indicate that PipX is a temperature-sensitive modulator, bringing new light to the study of environmental signaling in cyanobacteria. Our results also illustrate the enormous potential of the NanoBiT complementation system to fuel understanding of the mechanisms allowing cyanobacteria to initially respond and/or acclimatize to environmental factors.

## 1 Introduction

Cyanobacteria, phototrophic organisms that perform oxygenic photosynthesis, constitute an ecologically important phylum that is responsible for the evolution of the oxygenic atmosphere. They are the main contributors to marine primary production (Blank and Sánchez-Baracaldo, 2010; Lee et al., 2021) and are also ideal production systems for several high-value compounds (Khan and Fu, 2020; Lee et al., 2025). Cyanobacteria have developed sophisticated regulatory systems to adapt to challenging environmental conditions, including strategies to maintain the carbon/nitrogen balance [reviewed by Zhang C. C. et al. (2018) and Forchhammer and Selim (2020)]. To achieve this homeostasis, the signal transduction protein PII regulates the activity of proteins involved in nitrogen and carbon metabolism by direct protein–protein interactions (Forchhammer et al., 2022), perceiving metabolic information through the competitive binding of ATP or ADP and the synergistic binding of ATP and 2-oxoglutarate (2-OG) (Kamberov et al., 1995; Zeth et al., 2014). The global transcriptional regulator NtcA controls nitrogen assimilation in cyanobacteria (Herrero et al., 2001; Espinosa et al., 2006; Esteves-Ferreira et al., 2018) by also responding to the concentration of 2-OG, which provides a metabolic sensor of the carbon and nitrogen status.

**Figure 1:**
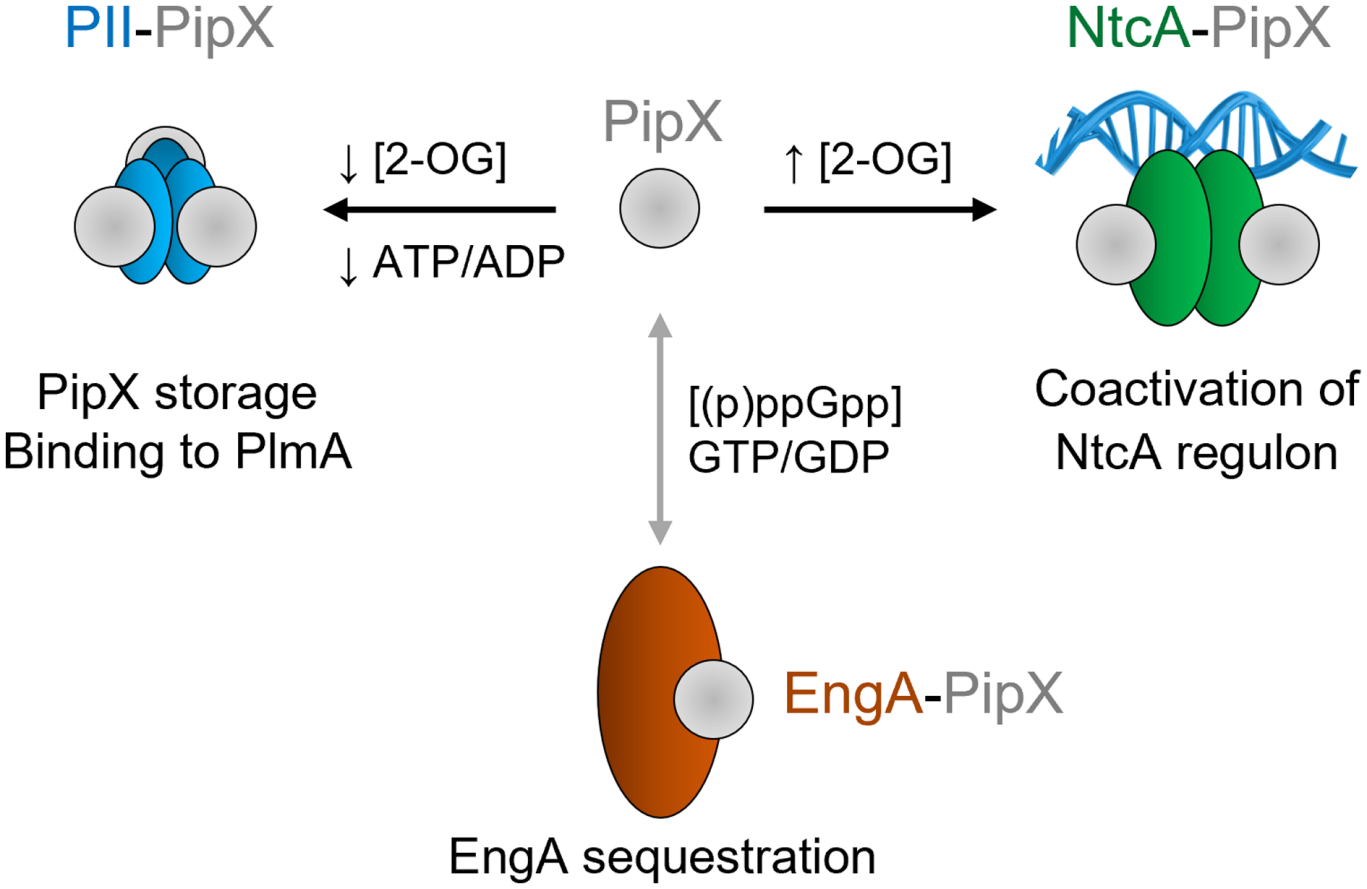
Functions and effectors of PipX complexes. Geometric representation of the indicated proteins in their corresponding oligomeric state, with area scaled according to the number of amino acids. Known or putative effectors involved in complex formation and their known functions are indicated. PipX–PII and PipX–NtcA complexes are oppositely regulated in response to 2-OG levels, while the PipX–PII complex also respond to the ATP/ADP ratio. PipX-EngA complexes would be modulated by EngA effectors, although the details concerning the effects of (p)ppGpp and of the GTP/GDP ratio are still unknown (indicated by a doble headed arrow, colored grey).

The PipX protein (Labella et al., 2020), identified by its ability to form complexes with PII and NtcA (Burillo et al., 2004; Espinosa et al., 2006, 2007, 2018; Llácer et al., 2010; Laichoubi et al., 2011; Jerez et al., 2024), is a unique and highly conserved protein exclusive to cyanobacteria. Regulation of protein–protein interactions with PipX is dependent on ligand binding by its partners (Figure 1). PII stabilizes PipX in *Synechococcus elongatus* PCC 7942 (hereafter *S. elongatus*) (Llop et al., 2023c). The binding of PipX to PII or NtcA is antagonistically tuned by 2-OG levels (Espinosa et al., 2006; Llácer et al., 2010; Zhao et al., 2010b). PipX uses the same surface from its TLD (Tudor-like domain)/KOW domain to bind to either 2-OG-bound NtcA, stimulating DNA binding and transcriptional activity, or to 2-OG-free PII; thus, PII sequestration of PipX at low 2-OG reduces the expression of NtcA-dependent gene targets (Espinosa et al., 2009, 2010, 2014; Zhao et al., 2010a; Laichoubi et al., 2012; Forcada-Nadal et al., 2018). PipX stabilizes the conformation of NtcA that is transcriptionally active and helps local recruitment of RNA polymerase (Forcada-Nadal et al., 2025) in response to nitrogen limitation (Espinosa et al., 2014; Giner-Lamia et al., 2017). PipX also interacts with the essential ribosome-assembly GTPase EngA (YphC/Der/YfgK) (Jerez et al., 2021). In *S. elongatus*, PipX interferes with EngA function under environmentally relevant conditions such as cold or light stress (Jerez et al., 2021; Llop et al., 2023a) and also interacts with the transcriptional regulator PlmA (Labella et al., 2016).

Temperature is a highly important environmental parameter for cyanobacteria with a considerable impact on both physiology and gene expression. Previous studies have focused on identifying regulators and gene targets of signal pathways involved in transcriptional regulation in response to either cold or heat shock (Sinetova and Los, 2016; Kobayashi et al., 2017; Mironov et al., 2019), two stresses that are sensed by different mechanisms in bacteria (Kusukawa and Yura, 1988; Weber and Marahiel, 2003; Shivaji and Prakash, 2010; Schumann, 2016; Yura, 2019; Zhang and Gross, 2021). An alternative approach, studying the *in vivo* effect of temperature shifts on regulatory protein complexes is now possible using the NanoBiT complementation system (Dixon et al., 2016), which is based on reconstitution of the small and high output bioluminescence enzyme NanoLuc. The NanoBiT system has been used in both mammalian (Kashima et al., 2021; Pipchuk and Yang, 2021; Sicking et al., 2021) and bacterial cells (Oliveira Paiva et al., 2019; Westerhausen et al., 2020; Rozbeh and Forchhammer, 2021, 2024; Bardelang et al., 2023; Jerez et al., 2024) to demonstrate the specificity of protein interactions of interest in their natural environment. Importantly, we have used it to demonstrate the opposing regulation of PipX-PII and PipX-NtcA complexes in real time in response to different nitrogen sources or to decreases in ATP levels in *S. elongatus* (Jerez et al., 2024). These studies showed that all three tagged derivatives were functional, highlighting the advantages of the NanoBit system in determining real time effects of ligands on complex formation and the competition for PipX between the two nitrogen regulators under environmentally relevant conditions for cyanobacteria.

Since temperature appears to be highly relevant for EngA function and interactions with PipX (Jerez et al., 2021; Llop et al., 2023a), we have used the NanoBit system to investigate the importance of temperature on the PipX interaction network *in vivo*. Previously, NanoLuc has been successfully used in thermal shift assays (Dart et al., 2018), with the luciferase signal remaining stable *in vitro* even at low (10 °C) and high (65 °C) temperatures (Hall et al., 2012; Ahmed et al., 2024). The NanoBiT complementation system has also been described as robust to temperature *in vivo* in the range from 20 to 50 °C (Dixon et al., 2016), retaining enzymatic activity across a wide thermal window (Melanie Dart et al., 2020). In addition, in *Synechocystis*, a NanoLuc-GFP fusion protein exhibited no signal variation between 15 and 30 °C (Nakamura et al., 2021).

Taking advantage of the NanoBit complementation system, we report here on the effects, in real time, of temperature shifts on protein complexes belonging to the paradigmatic PipX interaction network of cyanobacteria. We demonstrate that temperature regulates not only EngA levels and PipX-EngA complex formation but also the stability of PipX-PII and PipX-NtcA complexes in *S. elongatus*. Real-time experiments using the NanoBiT complementation system revealed the involvement of PipX in both early and late responses to temperature. This study illustrates the enormous potential of the NanoBiT complementation system to fuel our understanding of molecular mechanisms allowing cyanobacteria to initially respond and acclimatize to environmental conditions.

## 2 Materials and methods

### 2.1 Plasmid construction

The plasmids and primers used in this study are listed in Table 1; Supplementary Table S1, respectively. *Escherichia coli* XL1-Blue was used to perform Gibson assembly cloning (Gibson et al., 2009). All constructs were verified by automated Sanger sequencing.

**Table 1:** Plasmids.

Plasmid	Description, relevant characteristics	Source or reference
pUAGC87	*(lacI P_trc_^Osym^:engA)* into *NS3,* Nt^R^	Llop et al. (2023a)
pUAGC908	*engA* replaced with *cat*, Ap^R^ Cm^R^	Jerez et al. (2021)
pUAGC1161	*(P_pipX_:pipX:FL:SmBiT P_glnB_:glnB:FL:LgBiT)* into *NSI,* Ap^R^ Sm^R^	Jerez et al. (2024)
pUAGC1163	*(P_pipX_:pipX:FL:SmBiT P_ntcA_:ntcA:FL:LgBiT)* into *NSI,* Ap^R^ Sm^R^	Jerez et al. (2024)
pUAGC1165	*(P_pipX_:pipX:FL:SmBiT P_engA_:engA:FL:LgBiT)* into *NSI,* Ap^R^ Sm^R^	This work

Ap, ampicillin; Sm, streptomycin; Cm, chloramphenicol; Nt, nourseothricin; R, resistance; cat, chloramphenicol acetyltransferase.

The plasmid pUAGC1165 was obtained by assembling fragments F1 and F2, as described (Jerez et al., 2024). Fragment F1, comprising the *engA* coding region and 162 bp upstream, was amplified by PCR from *S. elongatus* genomic DNA using primers EngA-FL-LgBit-R and SmBiT-EngA-F. Fragment F2 was amplified by PCR from pUAGC1161 using primers FL-LgBiT-4F and SmBiT-2R.

### 2.2 Cyanobacteria transformation and strain verification

The *S. elongatus* strains used in this study are listed in Table 2. Transformations were performed essentially as described (Taton et al., 2020), and allele replacement verified by PCR. The primer pairs used were NSI-1R/NS1-2R for NSI, PipX-L80Q-F/LgBit-NS-4R for *engA*: LgBiT, 2,340-For/2341-Rev for *engA* inactivation, and NS3-seq-1F/NS3-seq-1R for NS3.

**Table 2:** Strains.

Strain	Genotype, relevant characteristics	Source or reference
*E. coli* XL1-Blue	*recA1 endA1 gyrA96 thi-1 hsdR17 supE44 relA1* lac [F′ *proAB* lacI^q^Z∆M15 Tn*10* (Tet^R^)]	Bullock et al. (1987)
WT	Wild type *S. elongatus* PCC7942	Pasteur Culture Collection
3^N^Ptrc^Osym^-EngA	*NS3:(lacI P_trc_:engA)*, Nt^R^	Llop et al. (2023a)
3^N^Ptrc^Osym^-EngA/Δ*engA*	*NS3:(lacI Ptrc:engA) ΔengA*:*cat*, Nt^R^ Cm^R^	Llop et al. (2023a)
1^S^PipXSmBiT-PIILgBiT	*NSI:(P_pipX_:pipX: FL:SmBiT P_glnB_:glnB:FL: LgBiT),* Sm^R^	Jerez et al. (2024)
1^S^PipXSmBiT-NtcALgBiT	*NSI:(P_pipX_:pipX:FL:SmBiT P_ntcA_:ntcA:FL:LgBiT),* Sm^R^	Jerez et al. (2024)
1^S^PipXSmBiT-EngALgBiT	*NSI:(P_pipX_:pipX:FL:SmBiT P_engA_:engA:FL:LgBiT),* Sm^R^	This work
1^S^PipXSmBiT-EngALgBiT/Δ*engA*	*NSI:(P_pipX_:pipX:FL:SmBiT P_engA_:engA:FL:LgBiT) ΔengA*:*cat,* Sm^R^ Cm^R^	This work

Tet, tetracycline; Sm, streptomycin; Cm, chloramphenicol; Nt, nourseothricin; R, resistance; cat, chloramphenicol acetyltransferase.

### 2.3 Cyanobacteria growth and culture conditions

*Synechococcus elongatus* cultures were routinely grown in blue–green algae BG11 medium [BG11_0_ supplemented with 17.5 mM sodium nitrate (NaNO₃) and 10 mM HEPES/NaOH (pH 7.8; Rippka et al., 1979)] at 30 °C under constant cool white fluorescent light, either in liquid cultures (150 rpm, 70 μmol photons m^−2^ s^−1^; mix of two clones) or on plates (50 μmol photons m^−2^ s^−1^; individual clones). When different temperature conditions were required, cultures grown under standard conditions in flasks were transferred to a Binder KBW 400 or Velp Scientifica™ FOC 2001 Connect incubator.

Solid media contained 1.5% (w/v) agar and, after autoclaving, were supplemented with 0.5 mM sodium thiosulfate (Na₂S₂O₃). When appropriate, antibiotics were added at the following concentrations: chloramphenicol (Cm, 3.5 μg/mL), streptomycin (Sm, 15 μg/mL), or nourseothricin (Nt, 15 μg/mL).

For liquid growth, cultures of 50 or 170 mL in BG11 were grown in baffled flasks. Growth was monitored by measuring the optical density at 750 nm (OD_750nm_) in 1 mL samples using an Ultrospec 2,100 Pro UV–Vis Spectrophotometer (Amersham Biosciences, Amersham, UK). All experiments were performed on mid-exponential phase cultures (OD_750nm_ = 0.4–0.8).

### 2.4 Protein extraction, immunodetection, and band quantification

For protein extraction, flask cultures grown under standard conditions were transferred to air incubators (Binder KBW 400 or Velp Scientifica™ FOC 2001 Connect) set to the relevant temperature. 10 mL samples were then harvested via 8 min centrifugation at 7300 × g, flash frozen in liquid nitrogen, and stored at −20 °C. The pellets were resuspended in 60 μL of lysis buffer [10 mM Tris/HCl pH 7.5, 0.5 mM EDTA, 1 mM *β*-mercaptoethanol, 1 mM phenylmethylsulfonyl fluoride (PMSF)], and cells were disrupted with 1 spoonful of 0.1 mm glass beads (≈30 μL), as described (Labella et al., 2016). Mixtures were subjected to three cycles of 60 s at a speed of 5 m/s in a high-speed homogenizer Minibeadbeater, followed by 60 s at 4 °C. Samples were centrifuged (5,500 × g for 5 min), and the supernatant fractions (crude protein extracts) were transferred to a new tube. Protein concentrations were estimated via the Bradford method (Bradford, 1976) using the PierceTM detergent-compatible Bradford assay kit (ThermoScientific, Waltham, MA, USA) on a VICTOR3TM 1,420 Multilabel Plate Reader. Crude protein extracts were stored at −20 °C until needed.

For immunodetection, 10–60 μg of total protein extracts were loaded into a sodium dodecyl sulphate polyacrylamide gel (SDS-PAGE; 15% polyacrylamide). Electrophoresis was followed by immunoblotting onto 0.2 μm polyvinylidene fluoride membranes (from GE Healthcare Technologies, Inc., Chicago, IL, USA), and the membranes were subsequently blocked with Tris-Buffered Saline (TBS-Tween; 20 mM Tris/HCl pH 7.5, 500 mM NaCl, Tween 20 0.1%) solution containing 5% non-fat dried milk for 1 h at room temperature and then incubated overnight in TBS-Tween with 2–5% non-fat dried milk with the corresponding primary antibody. Membranes were then incubated for 1 h at room temperature with a 1:150,000 dilution of ECL rabbit IgG and an HRP-linked F(ab’)2 fragment (from a donkey, GE Healthcare) or a 1:2,500 dilution of mouse IgG (from goat, Merck Millipore, Germany). The signal was detected using a SuperSignal WestFemto reagent (Thermo Fisher Scientific, Waltham, MA, USA) in a Biorad ChemiDoc Imager using the automatic exposure mode and avoiding pixel saturation. A 1:5,000 dilution of primary anti-PipX, anti-EngA, anti-PII, and anti-PlmA antibodies, or a 1:500 (EngA and NtcA) or 1:20,000 (PII) dilution of anti-LgBiT (Promega Corporation) antibody, were used separately.

### 2.5 NanoBiT bioluminescence assays

To measure NanoBiT bioluminescence, 500 μL aliquots of cyanobacterial cultures were transferred into luminometer tubes and were incubated in thermostatic water baths. At each timepoint samples were briefly vortexed with 10 μL of a fresh mQ water 13 μM solution of luciferin Q-108, prepared from Hikarazine-108 as described (Coutant et al., 2020) and incubated for 1 min under the same culture conditions. Bioluminescence was quantified using a luminometer (Junior LB9509, Berthold Technologies GmbH & Co. KG, Bad Wildbad, Germany) with a 5 s measurement time. Raw luminescence values were normalized by the OD_750nm_ of each culture.

500 μL aliquots of the cultures were transferred into 1.5 mL microcentrifuge tubes or 3.5 mL luminometer tubes for each timepoint and incubated in thermostatic water baths.

### 2.6 Intracellular ATP content determination

ATP extraction was essentially performed as described in Jerez et al. (2024). Briefly, 500 μL aliquots incubated in thermostatic water baths were flash-frozen in liquid nitrogen. ATP was extracted via three consecutive cycles of boiling (10 min, 100 °C) and freezing (liquid nitrogen), followed by centrifugation at 14,000 × g for 3 min at 4 °C. A 100 μL aliquot of the samples, or an appropriate dilution with mQ water if necessary, was mixed with 40 μL of a reaction solution containing 1 mM DTT, 0.25 mM luciferin, and 7.5 μg/mL luciferase from *Photinus pyralis*. The bioluminescence was measured in black 96-well microplates (OptiPlate-96\u00B0F HB; PerkinElmer, Waltham, MA, USA) using a VICTOR3TM 1,420 Multilabel Plate Reader (PerkinElmer, Waltham, MA, USA). The ATP content was quantified using the standard curve created in parallel.

### 2.7 Computational methods

Protein intensity levels were quantified from Western blot images using *ImageJ* v1.54g. Bands were selected using the “Rectangle” function, and their corresponding intensity profiles were measured with the “Wand” tool. Statistical analyses were performed using *RStudio* (RStudio: Integrated Development for R; RStudio, 2020).

PyMOL (The PyMOL Molecular Graphics System, Version 1.7.1.7, Schrödinger, LLC) was used to generate graphical representations of protein structures.

## 3 Results

### 3.1 EngA levels were downregulated by high temperature at the post-transcriptional level

**Figure 2:**
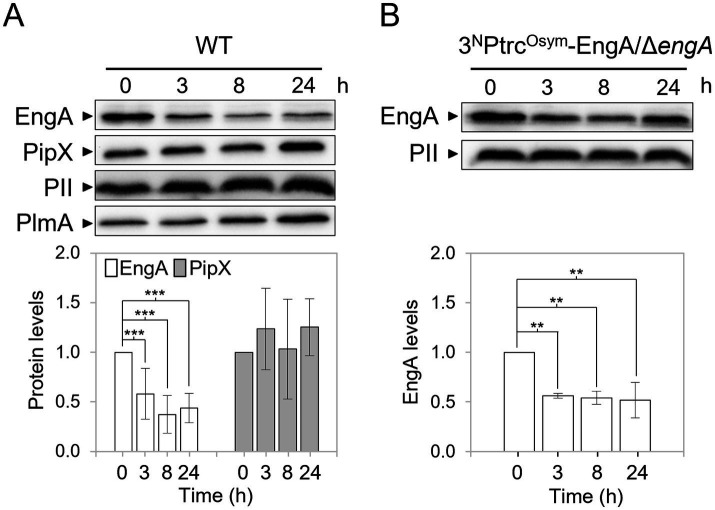
Effect of the temperature upshift on protein levels in *S. elongatus*. **(A,B)** *Top* – Representative immunodetections of the indicated proteins in the WT **(A)** and 3^N^Ptrc^Osym^-EngA/*engA* **(B)** strains at 42 °C. *Bottom*—Relative protein levels normalized to PlmA **(A)** or PII **(B)** and referred to the timepoint 0. Data are presented as means with error bars (standard deviation) from 10 (EngA) and 5 (PipX) biological replicates in **(A)** and from 4 replicates in **(B)**. A linear mixed model was performed with time as a fixed effect and experiment as a random effect. Comparison of protein levels between timepoint 0 and the others were made using pairwise comparisons with Kenward-Roger adjusted degrees of freedom and Bonferroni correction. Significance levels were denoted as *p* ≤ 0.01 (**) and *p* ≤ 0.001 (***).

We recently showed that the levels of the ribosome-assembly GTPase EngA increase after transfer from 30 °C to 18 °C while PipX or PII levels remain constant (Llop et al., 2023a). To investigate whether temperature upshift also affected EngA levels, we determined EngA, PipX and PII levels at different timepoints after transferring cultures from 30 °C to 42 °C. Western blot analysis with anti-EngA or anti-PipX antibodies were performed at different timepoints after transferring *S. elongatus* cultures from 30 °C to 42 °C. The immunodetection signal for the previously analysed transcriptional regulator PlmA (Labella et al., 2016) was first used as an internal control to normalize signals and determine protein levels at 42 °C for each protein. As additional control, we determined PII levels in parallel, showing that they were also indistinguishable amongst the different timepoints. Both PII and PlmA proteins are thermostable (Llop et al., 2023a; this work) and antibodies directed against these proteins showed minimal non-specific signal. As shown in Figure 2A; Supplementary Figure S1A, EngA levels were significantly lower at 42 °C, decreasing to less than 50% of the 30 °C level. The significant decrease in EngA levels, detected 3 h after the upshift, was maintained for 24-h. In contrast, PipX levels remained constant after the temperature upshift.

To gain insights into the mechanism involved in down regulation of EngA levels at 42 °C, we next generated the *S. elongatus* strain 3^N^Ptrc^Osym^-EngA/*ΔengA* (Table 1), where expression of the ectopic *engA* gene (allele *Ptrc^Osym^:engA*) is driven from an IPTG inducible promoter while coding sequences at the native *engA* locus were precisely replaced by the *cat* (chloramphenicol-acetyltransferase) gene (allele *engA:cat*). Cultures of this *S. elongatus* strain were transferred from 30 °C to 42 °C and extracts taken at different timepoints were subsequently analysed by Western blotting. As shown in Figure 2B; Supplementary Figure S1B, EngA levels decreased in strain 3^N^Ptrc^Osym^-EngA/*ΔengA* at 42 °C, indicating that cis-acting sequences upstream of the *engA* gene were not required for the observed downregulation in response to high temperature, and thus it implies RNA stability, post-transcriptional regulation or protein degradation.

In combination with our previous experiments performed at 18 °C (Llop et al., 2023a), it is evident that overall, EngA levels decreased in *S. elongatus* in response to temperature transitions to warmer environmental conditions, consistent with the role of EngA as an rRNA chaperone at low temperatures (Bharat and Brown, 2014). However, it is evident that the mechanistic response to temperature stress was different, since temperature downshift induced transcriptional regulation of EngA expression, whereas the heat shock response involves post-transcriptional control of EngA levels. This raises the question of whether the levels of other proteins acting as RNA chaperones were also finely tuned in response to temperature in cyanobacteria.

### 3.2 Generation of a NanoBiT reporter strain for PipX-EngA interactions in *S. elongatus*

**Figure 3:**
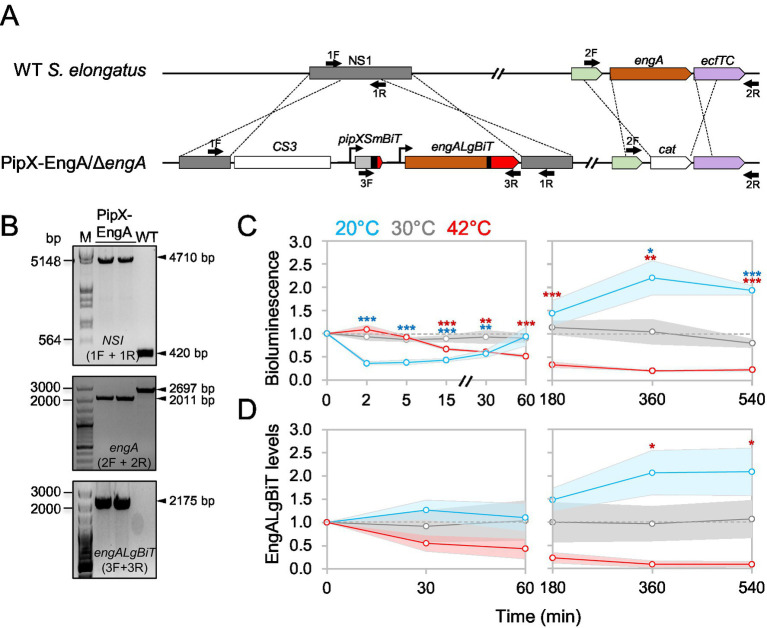
Construction and validation of a PipX-EngA NanoBit reporter and its response to temperature shifts in *S. elongatus.* **(A)** Schematic representation of NSI and *engA* regions at the *S. elongatus* chromosome from WT (top) or reporter strain (botton) showing *pipXSmBiT* and *engALgBiT* gene fusions and *CS3* and *cat* selection markers. The color code for *pipX* and *engA* genes is the same used for the proteins in Figure 1. Black bars indicate flexible linkers. **(B)** PCR analysis with the primers indicated as black arrows in *A*. M: *λ* HindIII/EcoRI or 100 bp size marker. **(C)** Bioluminescence signals obtained using Hikarazine-108 (CNRS) as the luciferase pro-substrate. Signals were referred to the timepoint 0, in cultures at 20 °C, 30 °C, or 42 °C corresponding to timepoints taken up to one (*left*) or 9 h (*right*). The time axis has been interrupted to improve data visualization. **(D)** EngALgBiT levels, normalized to PII and referred to timepoint 0, corresponding to timepoints taken up to one (*left*) or 9 h (*right*). Dashed grey lines mark the threshold at 1 in the graphs. Data are presented as means with error bars (standard deviation as shadows behind the lines) from at least three biological replicates. Welch’s *t*-test with Bonferroni correction was used to compare data between 30 °C and either 20 °C or 42 °C at the same timepoint. Significance levels were denoted for the corresponding color of the condition as *p* ≤ 0.05 (*), *p* ≤ 0.01 (**), and *p* ≤ 0.001 (***).

To determine the effect of temperature on PipX-EngA complex formation in *S. elongatus* we designed a PipX-EngA reporter construct guided by previously validated PipX-PII and PipX-NtcA reporters (Jerez et al., 2024). This construct expresses PipX-SmBiT and EngA-LgBiT fusion proteins from a neutral site in the *S. elongatus* chromosome (Figure 3A). To conserve wild type regulation, the upstream regulatory sequences of *pipX* and *engA* were also included. Introduction of the PipX-EngA reporter construct into the neutral site I (NSI) by allelic replacement was facilitated by a streptomycin-resistant marker cassette (C.S3) also included within the NSI insertions.

The PipX-EngA reporter plasmid (pUAGC1165, Table 2) was introduced into *S. elongatus*, and independent streptomycin-resistant transformants were PCR-analysed to confirm complete segregation of the modified NSI alleles in *S. elongatus*. Validated clones were selected for further work (strain 1^S^PipXSmBiT-EngALgBiT; Figure 3B, upper panel).

Since *engA* is essential in *S. elongatus*, we next tested the functionality of the EngA-LgBiT fusion protein by its ability to provide EngA essential functions. To this end, *S. elongatus* strain 1^S^PipXSmBiT-EngALgBiT was used to inactivate the native *engA* gene by allelic replacement with the *engA:cat* derivative. The corresponding alleles are illustrated in Figure 3A. Complete segregation of the null allele indicated complementation of EngA essential functions by EngA-LgBiT. Validated 1^S^PipXSmBiT-EngALgBiT *engA* clones bearing *engALgBiT*, instead of the wild type *engA* allele (Figure 3B, middle and lower panels), were selected and referred to hereafter as the PipX-EngA reporter strain.

### 3.3 PipX-EngA complexes were transiently impaired by temperature downshift, but increased in cultures acclimatized to low temperature

To test the effect of temperature changes on PipX-EngA complexes in real-time we measured bioluminescence at different timepoints after shifting cultures of the corresponding NanoBiT reporter strain from 30 °C to 20 °C or 42 °C. Luminescence values were also recorded from control samples maintained at 30 °C. To facilitate the distinction between the initial responses to cold or heat stress from longer term acclimatization responses, we split data from the same experiments into independent figures to represent “early” (up to 60 min, Figure 3C left) and “late” (up to 9 h, Figure 3C right) timepoints.

Given that EngA levels were temperature-dependent in wild type *S. elongatus* (Llop et al., 2023a; Figure 2), it was important to consider the contribution of these changes to the bioluminescence signals from NanoBit reporters. Therefore, samples were taken at several of the timepoints used for luciferase assays and subsequently analysed by Western blot with anti-LgBiT (Figure 3D; Supplementary Figures S1C, S2A).

Transfer to 20 °C produced an extremely rapid decay of the bioluminescence signal (by about 70% at the 2 min timepoint), indicating that temperature downshift dissociated PipX-EngA complexes. In contrast, transfer to 42 °C resulted in a very slow reduction of the PipX-EngA signal that mirrored the decrease in EngALgBiT levels.

The fast decay of the bioluminescence signal upon transfer to 20 °C was fully recovered at the 60 min timepoint, with signals continuing to increase and reaching maximal values at the longest timepoints of the experiment, where they closely correlated with EngALgBiT levels (Figures 3C,D). This implies that the immediate response to temperature downshift resulted in complex dissociation, whereas acclimatization allowed reestablishment of complexes in the longer term, in agreement with the increase in EngA levels.

The response of complexes to heat shock was in clear contrast to that obtained upon transfer to 20 °C. Rapid or transient changes were not observed and there was a close correlation between bioluminescence signals and the EngALgBiT levels observed between all timepoints taken from cultures at 42 °C.

Considering the relative bioluminescence values under steady-state conditions (longer term exposure to temperature), it follows that a larger number of PipX-EngA complexes were formed in cultures exposed to cold rather than to heat stress. The increase of the number of PipX-EngA complexes during cold stress may facilitate down tuning EngA activity at low temperature (Jerez et al., 2021; Llop et al., 2023a).

### 3.4 PipX-PII and PipX-NtcA complexes were rapidly impaired by temperature downshift, but responded differently to heat shock

**Figure 4:**
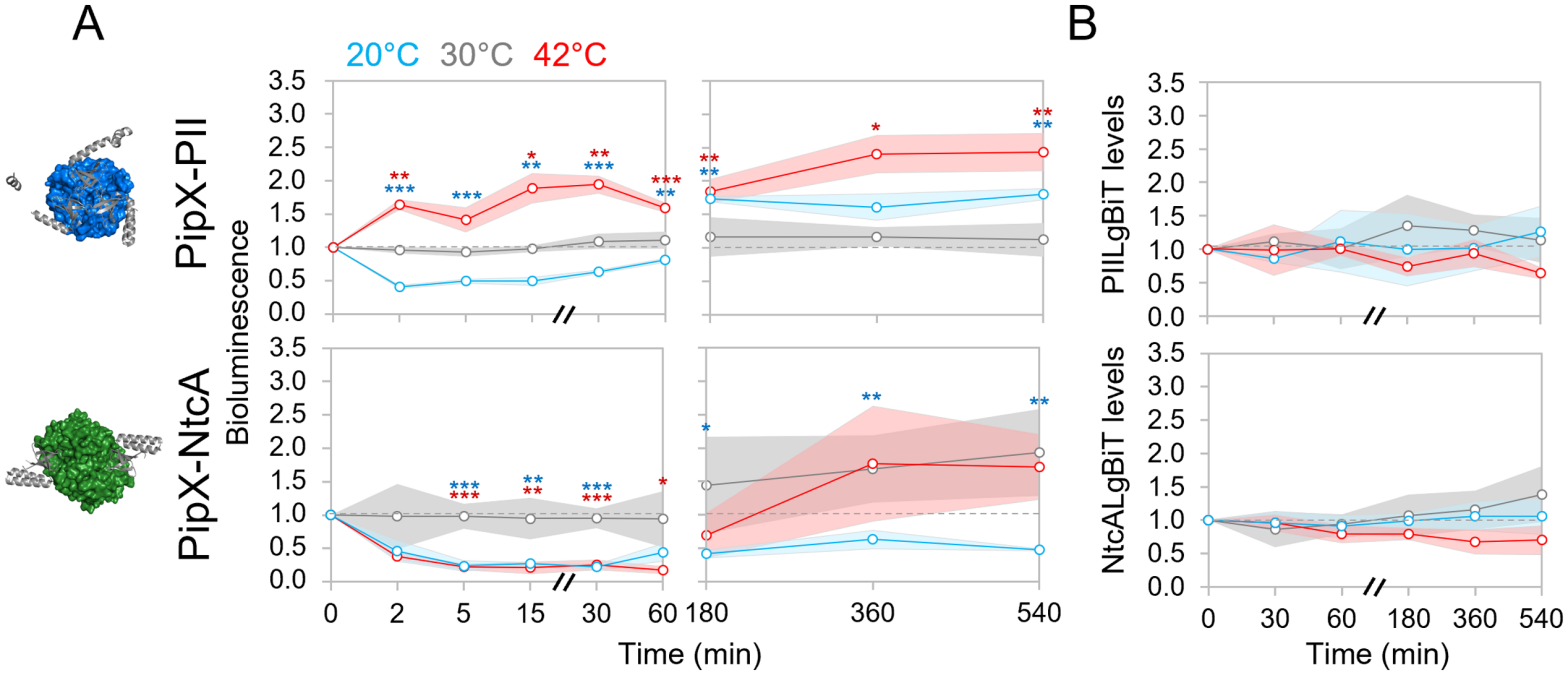
Regulation of PipX complexes in response to temperature up or downshifts. **(A)** Bioluminescence signal, referred to timepoint 0, in *S. elongatus* cultures at 20 °C, 30 °C, or 42 °C corresponding to timepoints taken up to 1 (*left*) or 9 hours (*right*). **(B)** Protein levels normalized to PlmA (for PIILgBiT) or PII (for NtcALgBiT) and referred to the timepoint 0. PipX (grey; ribbon) in complex with PII (blue; surface; PDB: 2XG8) or NtcA (green; surface; PDB: 2XKO) is shown at the left. Other details as in Figure 3.

PipX-PII and PipX-NtcA complexes have been extensively studied as part of a protein network for metabolic regulation and signaling of the intracellular carbon/nitrogen and energy status, but so far not in other contexts that may also be relevant to the PipX interaction network. We have previously used the NanoBit system to study the regulation of PipX-PII and PipX-NtcA complexes in real time in *S. elongatus* (Jerez et al., 2024). Here, and similarly to the analysis of PipX-EngA complexes, we utilized the Nanobit system to determine the effect of temperature changes on PipX-PII and PipX-NtcA complexes at different timepoints after shifting cultures from 30 °C to 20 °C or 42 °C (Figure 4A).

Western blot analyses with anti-LgBiT indicated that the levels of both PIILgBiT and NtcALgBiT remained relatively constant during the time course of the experiment (Figure 4B; Supplementary Figures S1D, S2B,C). Thus, differences in the bioluminescence signal after the temperature shifts should directly report the *in vivo* dynamics of PipX-PII and PipX-NtcA complexes in response to cold or heat shock.

Changes in bioluminescence signals took place rapidly (within 2 min) after each of the two temperature shifts (Figure 4A). As in the case of the PipX-EngA interaction (Figure 3C), temperature downshift decreased bioluminescence signals from both PipX-PII and PipX-NtcA reporters, indicating that a common mechanism could be involved in signaling temperature downshift to all three PipX complexes studied in this work. In view of this similar response, it is unlikely that the changes in the concentration or the presence of well-known ligands (ATP/ADP ratio or the 2-OG concentration), that affect partner switching of PipX between PII and NtcA (Figure 1), were involved.

In contrast, heat shock triggered opposite responses for PipX-PII and PipX-NtcA complexes, respectively increasing or decreasing complex formation, reminiscent of partner switching.

### 3.5 PipX-PII complexes were abundant during adaptation to both cold and heat stress

At 20 °C the initial temperature downshift-promoted dissociation of PII-PipX complexes slowly reversed and between the 3 h and 9 h timepoints, bioluminescence signals were maintained above the control levels at 30 °C, while PipX-NtcA signals remained minimal (Figure 4A). The contrasting behavior of PipX-PII and PipX-NtcA complexes after acclimatization to low temperatures suggests that binding of PipX to the highly abundant protein PII was favored to the detriment of PipX-NtcA complexes. It is therefore likely that the NtcA regulon was down regulated as part of the acclimatization response to low temperature stress.

The initial increase in PipX-PII complex formation at 42 °C remained above the control levels at 30 °C during the 9 h’ time course suggesting a role for PipX-PII complexes in acclimatization to heat stress. On the other hand, bioluminescence signals from the PipX-NtcA reporter were highly variable towards the end of the experiment at both 42 °C and 30 °C and thus, they were not easily rationalized. However, the signals from the PipX-NtcA complexes at 20 °C were more consistent during the later stages of the time course, congruent with a model in which modulation of the NtcA regulon is necessary for temperature adaptation as noted above.

## 4 Discussion

Temperature is a very important environmental parameter for most biological processes that directly impacts on the structure and function of different cellular components with thermodynamically sensitive proteins. Responses to temperature shifts have been widely investigated in cyanobacteria and other bacterial groups. So far, the main experimental approaches to unravel the molecular details involved have focused on identifying regulatory proteins triggering a transcriptional response and on their gene targets. The former includes two-component systems where a sensor histidine kinase detects membrane fluidity and transmits the signal to its cognate partners by phosphorylation (Weber and Marahiel, 2003; Shivaji and Prakash, 2010; Schumann, 2016; Sinetova and Los, 2016; Kobayashi et al., 2017; Mironov et al., 2019; Yura, 2019; Zhang and Gross, 2021), although additional complexity is emerging from those studies (Los et al., 2010). In this work, we used a different but complementary approach to gain insights into the mechanisms by which temperature can be sensed and modulate signaling pathways. Taking advantage of the NanoBit complementation system, we report here on the effects, in real time, of temperature shifts on protein complexes belonging to the paradigmatic PipX interaction network of cyanobacteria.

**Figure 5:**
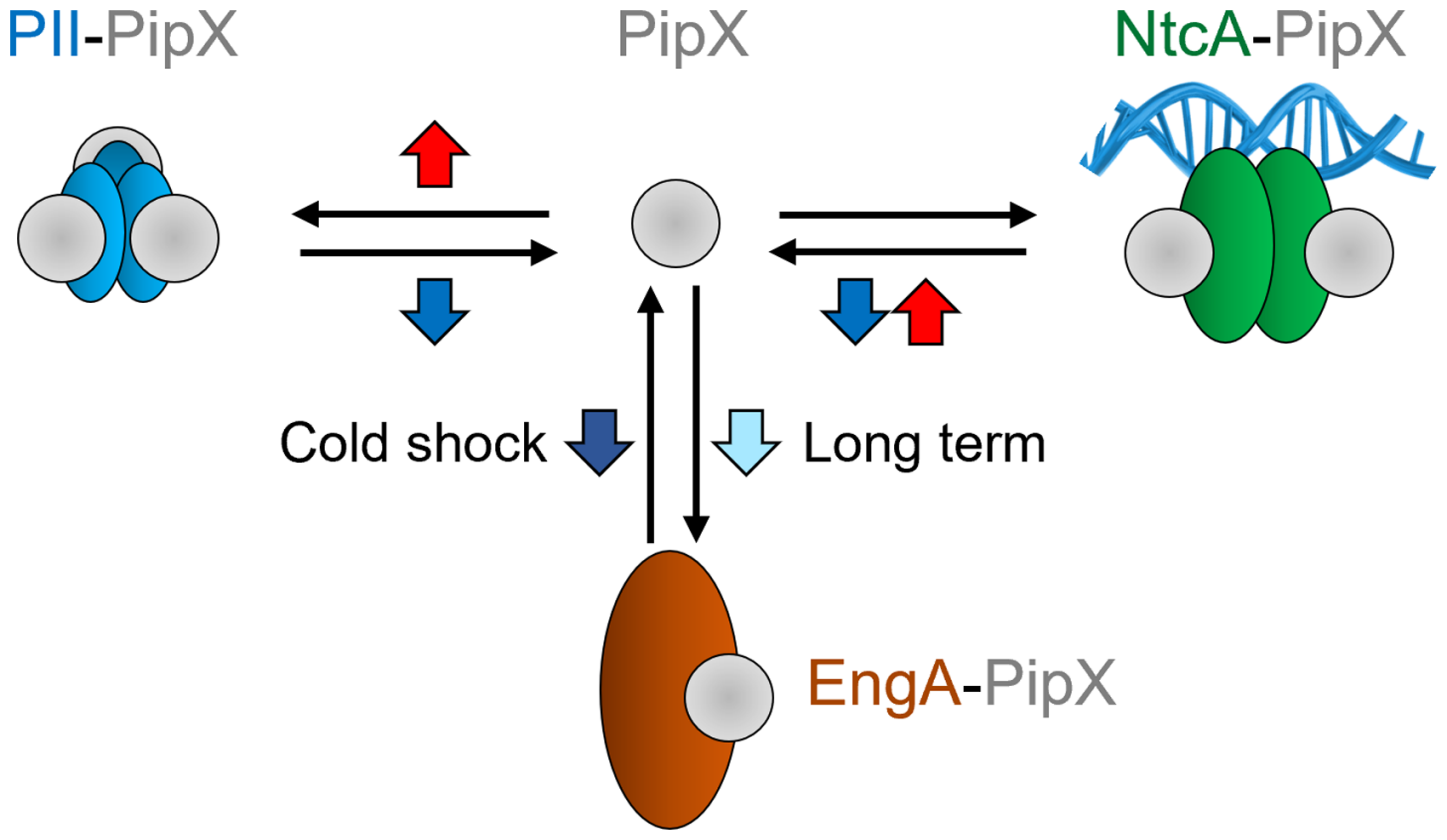
Effect of temperature shifts on PipX complexes. Blue down and red up arrows schematically summarize significant effects due to temperature switches from 30 °C to 20 °C or 42 °C, respectively. For all three complexes, changes favoring complex formation are indicated by the black arrow pointing towards the complexes. Conversely, temperature changes promoting complex dissociation are indicated by the arrows pointing towards PipX. For PipX-EngA the early response to cold shock promotes dissociation of the complex (indicated by a dark blue arrow). However, long term acclimatization to cold promotes complex formation (light blue arrow). Other details as in Figure 1.

We have shown here that the PipX interaction network was highly influenced by temperature, which could act at different regulatory levels to alter the distribution of PipX into different protein complexes. The interaction analysis presented in this work is schematically summarized in Figure 5, with all three complexes responding in different ways to temperature up (red arrows) or downshifts (blue).

In the case of EngA, long-term adaptation to a different temperature resulted in significant changes in the signal for PipX-EngA complexes that reflected the temperature-dependence of EngA levels, which were higher at low than at high temperatures. In contrast, changes observed during acclimatization to temperature by PipX-PII or PipX-NtcA complexes did not depend on the levels of the partners involved, which remained constant, and here the effect was likely to be attributed to altered levels of the ligands modulating complex formation, further suggesting that temperature shifts trigger metabolic changes affecting the relevant ligands. Although 2-OG levels were not investigated here and we found no significant differences between the intracellular levels of ATP from *S. elongatus* cultures at 30 °C or 42 °C (Supplementary Table S2), the results were still in agreement with the involvement of metabolic effector ligands in signaling.

In contrast to the long-term adaptation to temperature, the immediate response of PipX-PII, PipX-NtcA and PipX-EngA complexes to temperature shifts was rapid (and in some cases transient), corresponding to fast changes in affinity that, although equilibrate towards a steady state during adaptation to temperature stress, cannot be attributed to changes in the levels of the interacting proteins. Importantly, the significant impairment of PipX-PII, PipX-NtcA and PipX-EngA complexes after temperature downshift cannot be easily explained based on the known effectors of these proteins.

It is worth noting that PII and PII-like proteins can bind and be regulated by different types of nucleotides including the second messenger’s cyclic adenosine monophosphate (cAMP) and cyclic di-adenosine monophosphate (3′,5’-cAMP) (Selim and Alva, 2024). Nucleotides (p)ppGpp, cyclic di-GMP and 2′,3’-cAMP have been shown to be involved in signalling temperature downshift in Gram positive bacteria or plants (Gupta et al., 2016; Boniecka et al., 2017). In addition, EngA proteins also bind to (p)ppGpp (Corrigan et al., 2016; Zhang Y. et al., 2018; Wang et al., 2019; Mehrez et al., 2023). Unfortunately, cold is not one of the stress conditions that have been investigated in connection with the stringent response and (p)ppGpp levels in cyanobacteria (Hood et al., 2016; Puszynska and O’Shea, 2017; Llop et al., 2023b), and we cannot safely rule out the involvement of additional ligands at this stage.

In the light of the importance of temperature for gene expression and cell physiology, finding that temperature shifts affected PipX partner interactions is not very surprising. The changes observed after several minutes to hours would mainly impact metabolism, gene expression and ribosome assembly acting via PII, NtcA and EngA, respectively. However, decreased interactions of PipX with those partners upon a sudden drop of temperature is likely to favor the binding of PipX to other, yet unknown, partners.

PipX appears to act as a growth brake under stress conditions or when overexpressed, inhibiting relevant processes including photosynthesis (Labella et al., 2017; Jerez et al., 2021; Llop et al., 2023a,b) and EngA-dependent ribosome assembly/translation during cold or high light stress (Jerez et al., 2021; Llop et al., 2023a). Since temperature downshift decreases photosynthesis (Jansz and Maclean, 1973; Mackey et al., 2013) and triggers photodamage because the rate of electron consumption decreases while the light collected by the photosystems remains the same (Imlay, 2003), it is tempting to propose that photosynthesis-related protein(s) may be involved in the change of partners inferred for PipX upon temperature downshift.

Different experimental approaches such as co-localisation with the RNA-protein complexes involved in transcription, RNA metabolism and transcription initiation (Riediger et al., 2021) as well as interactions with the sigma and gamma subunits of the RNA polymerase (Forcada-Nadal et al., 2025), support the multifunctionality of PipX. Given that the *pipX* gene is a hallmark of cyanobacterial genomes, it is tempting to propose that PipX forms part of an early protective response to rapidly decrease photosynthesis and growth upon temperature downshift.

Since the affinity of the binding reactions is temperature-dependent and in general, dissociation of protein complexes is slower at low temperatures, the rapid dissociation of PipX complexes after temperature downshift suggests that PipX complexes exhibit cold sensitivity. Furthermore, the exhaustively mapped high dynamics of the protein in the nanosecond-to-picosecond time regime at physiological pH (Forcada-Nadal et al., 2017) agrees with a high potential of PipX for conformational changes. Our working hypothesis is that PipX must suffer local or global folding events for very short periods of time that may contribute to modulate its different interactions.

Finaly, this work calls attention to the importance of performing real time experiments to study the regulation of protein complexes in response to environmentally relevant changes and anticipates further breakthroughs in our understanding of signaling networks.

## Data Availability

The original contributions presented in the study are included in the article/Supplementary material, further inquiries can be directed to the corresponding author.
