# Supplementary figures and images for: Temperature changes are signaled in cyanobacteria through the PipX interaction network
Source: Front Microbiol. 2025 Nov 26;16:1688974. doi: 10.3389/fmicb.2025.1688974 (PMC12693397; doi:10.3389/fmicb.2025.1688974)

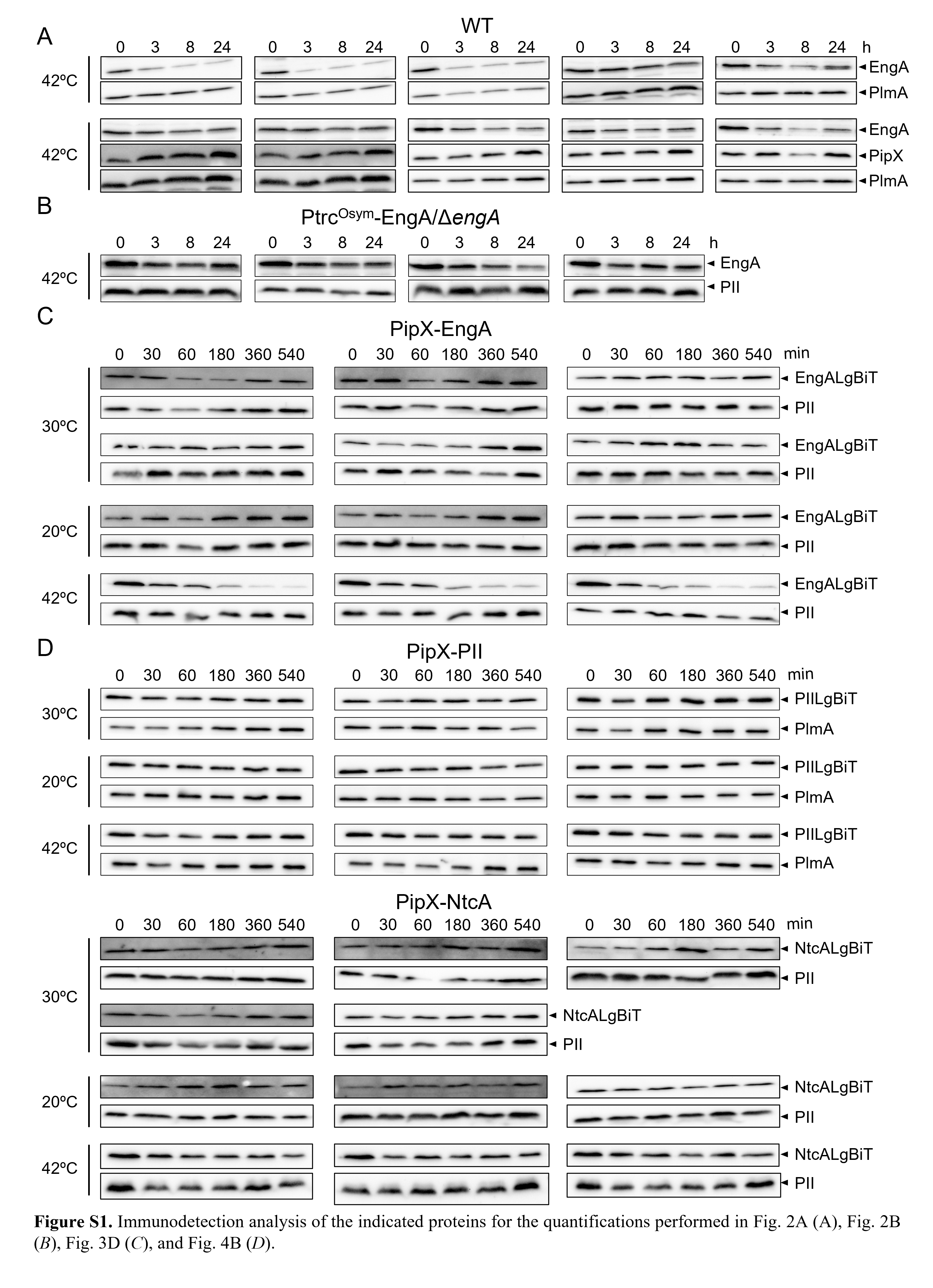

Supplement: Supplementary file 1 [file Image_1.TIF]

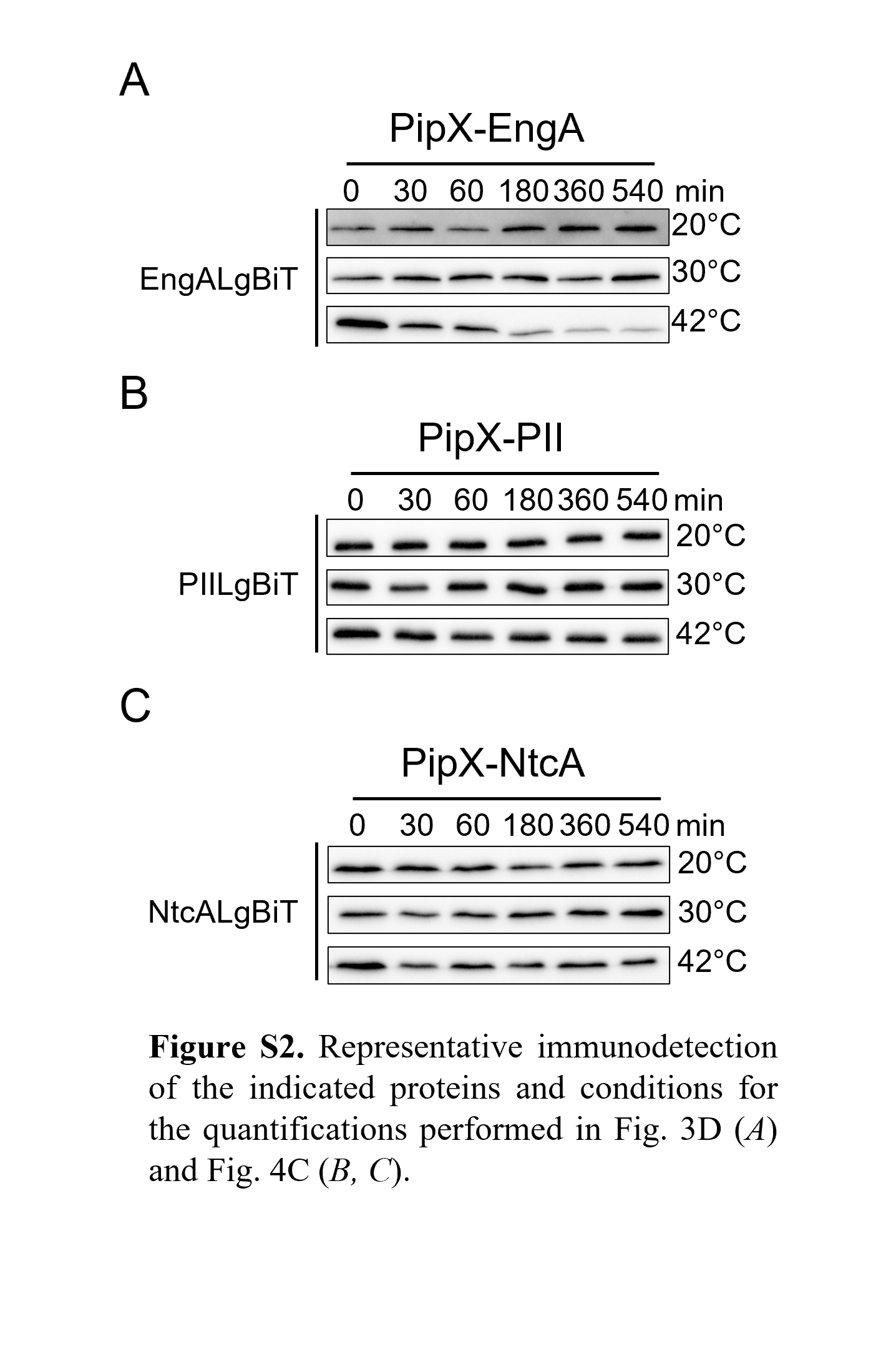

Supplement: Supplementary file 2 [file Image_2.TIF]
