# Supplementary material for: Temperature changes are signaled in cyanobacteria through the PipX interaction network
Source: Front Microbiol. 2025 Nov 26;16:1688974. doi: 10.3389/fmicb.2025.1688974 (PMC12693397; doi:10.3389/fmicb.2025.1688974)
Supplement: Supplementary file 3 [file Table_1.DOCX]

**Table S1.** Oligonucleotides.

| **Name** | **Sequence (5'— 3'**) |
| --- | --- |
| EngA-FL-LgBit-R | TCCGCTACTTCCGCCGCCTCCGCTAACGCGAGTCGCTCGATTG |
| SmBiT-EngA-F | CCGGCTGTTCGAGGAAATCCTGTAGTTCTGGGAGGCAGCAGTGC |
| FL-LgBiT-4F | CGGAGGCGGCGGAAGTAGCGGAGTGTTTACCCTGGAAGATTTC |
| SmBiT-2R | CTACAGGATTTCCTCGAACAGCCGGTAGCCGGTG |
| NS3-seq-1F | ACCTCCGGCAGTCAATTA |
| NS3-seq-1R | AGGGACTGGTTGATCGGT |
| NSI-1R | TGCCTGAAAGCGTGACGAGC |
| NS1-2R | CGGCCGAAAATGACAAGATC |
| PipX-L80Q-F | GCAGGAATACAACCAGCAGCAGCAAGTCTTCAAAC |
| LgBit-NS-4R | GAGGTCGACTCTAGAGGATCTTAGCTGTTGATGGTCACTCT |
| 2340-For | CCGAGGATCCTGATGTGACTGGCGC |
| 2341-rev | CAGAGTCGACGCCATTGACTGAGG |

**Table S2. ATP levels in response to temperatures up or downshifts.** Relative ATP levels, normalized with the OD_750_ and referred to timepoint 0, in cultures incubated at 20°C, 30°C, or 42°C for the indicated times. Data are presented as means with error bars (±standard deviation) from at least six (0-60 min) or three (180-540 min) biological replicates. Welch's t-test with Bonferroni correction was used to compare data between 30°C and either 20°C or 42°C at the same timepoint. Significant differences (p ≤ 0.05 (*)) were detected at the 5-min timepoint for 20°C.

| Temperature | Time (min) | | | | | | | | |
| --- | --- | --- | --- | --- | --- | --- | --- | --- | --- |
|  | 0 | 2 | 5 | 15 | 30 | 60 | 180 | 360 | 540 |
| 20°C | 1 | 0.88 ±0.07 | 0.72* ±0.08 | 0.76 ±0.16 | 0.82 ±0.14 | 0.80 ±0.20 | 0.72 ±0.12 | 0.73 ±0.11 | 0.72 ±0.04 |
| 30°C | 1 | 0.96 ±0.09 | 0.89 ±0.12 | 0.90 ±0.11 | 0.90 ±0.10 | 0.87 ±0.08 | 0.89 ±0.07 | 0.76 ±0.09 | 0.91 ±0.29 |
| 42°C | 1 | 0.94 ±0.19 | 1.00 ±0.23 | 1.05 ±0.28 | 0.93 ±0.19 | 0.83 ±0.24 | 0.81 ±0.12 | 0.87 ±0.02 | 0.81 ±0.10 |
